# Piperidinium 3-hydr­oxy-2-naphthoate

**DOI:** 10.1107/S1600536808025567

**Published:** 2008-08-13

**Authors:** Yong-Tao Wang, Gui-Mei Tang, Yong-Chun Zhang, Wen-Zhu Wan

**Affiliations:** a Department of Chemical Engineering, Shandong Institute of Light Industry, Jinan, Shandong 250353, People’s Republic of China

## Abstract

The crystals of the title salt, C_5_H_12_N^+^·C_11_H_7_O_3_^−^, were obtained from a methanol/water solution of 3-hydr­oxy-2-naphthoic acid and piperidine at room temperature. In the crystal structure, the piperidinium cations display a chair conformation and link with hydroxy­naphthoate anions *via* N—H⋯O and C—H⋯O hydrogen bonds. An intra­molecular O—H⋯O inter­action is also present.

## Related literature

For background, see: Shen *et al.* (2008 ▶); Wang *et al.* (2005*a* ▶,*b* ▶, 2006 ▶).
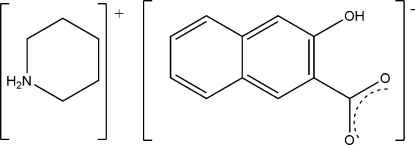


## Experimental

### 

#### Crystal data

C_5_H_12_N^+^·C_11_H_7_O_3_^−^
*M* *_r_* = 273.32
Monoclinic, 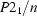

*a* = 8.6683 (3) Å
*b* = 19.4537 (5) Å
*c* = 9.5932 (3) Å
β = 111.959 (2)°
*V* = 1500.34 (8) Å^3^
*Z* = 4
Mo *K*α radiation
μ = 0.08 mm^−1^
*T* = 298 (2) K
0.40 × 0.30 × 0.20 mm

#### Data collection

Bruker SMART CCD area-detector diffractometer
Absorption correction: none
10640 measured reflections
3385 independent reflections
1512 reflections with *I* > σ(*I*)
*R*_int_ = 0.035

#### Refinement

*R*[*F* ^2^ > 2σ(*F* ^2^)] = 0.048
*wR*(*F* ^2^) = 0.160
*S* = 0.99
3385 reflections
181 parameters
H-atom parameters constrained
Δρ_max_ = 0.13 e Å^−3^
Δρ_min_ = −0.13 e Å^−3^

### 

Data collection: *SMART* (Bruker, 2001 ▶); cell refinement: *SAINT* (Bruker, 2001 ▶); data reduction: *SAINT*; program(s) used to solve structure: *SHELXTL* (Sheldrick, 2008 ▶); program(s) used to refine structure: *SHELXTL*; molecular graphics: *SHELXTL*; software used to prepare material for publication: *SHELXTL*.

## Supplementary Material

Crystal structure: contains datablocks global, I. DOI: 10.1107/S1600536808025567/xu2446sup1.cif

Structure factors: contains datablocks I. DOI: 10.1107/S1600536808025567/xu2446Isup2.hkl

Additional supplementary materials:  crystallographic information; 3D view; checkCIF report

## Figures and Tables

**Table 1:** Hydrogen-bond geometry (Å, °)

*D*—H⋯*A*	*D*—H	H⋯*A*	*D*⋯*A*	*D*—H⋯*A*
O1—H1*B*⋯O3	0.82	1.77	2.504 (2)	149
N1—H1*C*⋯O2^i^	0.96	1.83	2.783 (2)	173
N1—H1*D*⋯O3	0.96	1.75	2.709 (2)	173
C12—H12*A*⋯O1^ii^	0.97	2.40	3.336 (3)	161

Symmetry codes: (i) 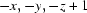; (ii) 
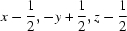
.

**Fig. 1.**
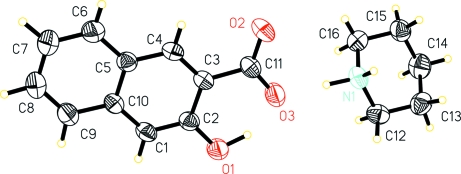
A drawing of (I), with the atom numbering scheme. Displacement ellipsoids are drawn at the 30% probability level.

## supplementary crystallographic information

### Comment

In some biological system, intermolecular interactions play the important role (Shen *et al.*, 2008), these interactions have attracted our much attention in past years. A series of compounds with weak intermolecular interactions have been synthesized and their crystal structures have been characterized (Wang *et al.*, 2005a,b, 2006). As part of our investigation, we recently prepared the title compound and present here its crystal structure.

The molecular structure of the title compound is shown in Fig. 1. The asymmetric unit contains one 3-hydroxy-2-naphthoate anion and one piperidinium cation. The piperidinium cation displays a typical chair conformation. The carboxylate group is coplanar with the naphthalene ring. Intermolecular N—H···O and C—H···O hydrogen bonding presents in the crystal structure (Table 1).

### Experimental

3-Hydroxy-2-naphthoic acid (94 mg, 0.5 mmol) and piperidine (43 mg, 0.5 mmol) were dissolved in methanol (5 ml) and water (1 ml) at room temperature. The single crystals of the title compound were obtained from the solution after several days.

### Refinement

H atoms were placed in calculated positions with O—H = 0.82, N—H = 0.96, C—H = 0.93 (aromatic) or 0.97 Å (methylene), and refined in riding mode with U_iso_(H) = 1.5U_eq_(O,N) and 1.2U_iso_(C).

### Crystal data

**Table:**

C_5_H_12_N^+^·C_11_H_7_O_3_^−^	*F*(000) = 584
*M**_r_* = 273.32	*D*_x_ = 1.210 Mg m^−^^3^
Monoclinic, *P*2_1_/*n*	Mo *K*α radiation, λ = 0.71073 Å
Hall symbol: -P 2yn	Cell parameters from 1627 reflections
*a* = 8.6683 (3) Å	θ = 2.5–20.5°
*b* = 19.4537 (5) Å	µ = 0.08 mm^−^^1^
*c* = 9.5932 (3) Å	*T* = 298 K
β = 111.959 (2)°	Block, colourless
*V* = 1500.34 (8) Å^3^	0.40 × 0.30 × 0.20 mm
*Z* = 4	

### Data collection

**Table:**

Bruker SMART CCD area-detector diffractometer	1512 reflections with *I* > σ(*I*)
Radiation source: fine-focus sealed tube	*R*_int_ = 0.035
graphite	θ_max_ = 27.4°, θ_min_ = 2.1°
φ and ω scans	*h* = −9→11
10640 measured reflections	*k* = −25→22
3385 independent reflections	*l* = −12→12

### Refinement

**Table:**

Refinement on *F*^2^	Primary atom site location: structure-invariant direct methods
Least-squares matrix: full	Secondary atom site location: difference Fourier map
*R*[*F*^2^ > 2σ(*F*^2^)] = 0.048	Hydrogen site location: inferred from neighbouring sites
*wR*(*F*^2^) = 0.160	H-atom parameters constrained
*S* = 0.99	*w* = 1/[σ^2^(*F*_o_^2^) + (0.0733*P*)^2^] where *P* = (*F*_o_^2^ + 2*F*_c_^2^)/3
3385 reflections	(Δ/σ)_max_ < 0.001
181 parameters	Δρ_max_ = 0.13 e Å^−^^3^
0 restraints	Δρ_min_ = −0.13 e Å^−^^3^

### Special details

**Table:**

Geometry. All e.s.d.'s (except the e.s.d. in the dihedral angle between two l.s. planes) are estimated using the full covariance matrix. The cell e.s.d.'s are taken into account individually in the estimation of e.s.d.'s in distances, angles and torsion angles; correlations between e.s.d.'s in cell parameters are only used when they are defined by crystal symmetry. An approximate (isotropic) treatment of cell e.s.d.'s is used for estimating e.s.d.'s involving l.s. planes.
Refinement. Refinement of *F*^2^ against ALL reflections. The weighted *R*-factor *wR* and goodness of fit *S* are based on *F*^2^, conventional *R*-factors *R* are based on *F*, with *F* set to zero for negative *F*^2^. The threshold expression of *F*^2^ > σ(*F*^2^) is used only for calculating *R*-factors(gt) *etc*. and is not relevant to the choice of reflections for refinement. *R*-factors based on *F*^2^ are statistically about twice as large as those based on *F*, and *R*- factors based on ALL data will be even larger.

### Fractional atomic coordinates and isotropic or equivalent isotropic displacement parameters (Å^2^)

**Table:**

	*x*	*y*	*z*	*U*_iso_*/*U*_eq_	
N1	−0.1726 (2)	0.09197 (8)	0.39856 (18)	0.0718 (5)	
H1C	−0.1979	0.0491	0.4346	0.108*	
H1D	−0.0626	0.1054	0.4635	0.108*	
O1	0.31760 (19)	0.22455 (7)	0.73435 (18)	0.0944 (5)	
H1B	0.2331	0.2051	0.6786	0.142*	
O2	0.24997 (18)	0.03674 (7)	0.51894 (15)	0.0832 (5)	
O3	0.14101 (19)	0.13370 (8)	0.56381 (18)	0.0948 (5)	
C1	0.6069 (3)	0.20988 (10)	0.8318 (2)	0.0692 (6)	
H1A	0.6176	0.2530	0.8763	0.083*	
C2	0.4523 (3)	0.18561 (9)	0.7481 (2)	0.0636 (5)	
C3	0.4321 (2)	0.12071 (9)	0.67629 (19)	0.0561 (5)	
C4	0.5718 (3)	0.08253 (9)	0.69633 (19)	0.0604 (5)	
H4A	0.5597	0.0398	0.6498	0.072*	
C5	0.7325 (3)	0.10544 (10)	0.7844 (2)	0.0629 (5)	
C6	0.8758 (3)	0.06627 (12)	0.8062 (3)	0.0930 (7)	
H6A	0.8654	0.0229	0.7626	0.112*	
C7	1.0287 (3)	0.09073 (16)	0.8897 (3)	0.1178 (10)	
H7A	1.1223	0.0642	0.9027	0.141*	
C8	1.0468 (3)	0.15586 (15)	0.9569 (3)	0.1102 (9)	
H8A	1.1524	0.1724	1.0137	0.132*	
C9	0.9119 (3)	0.19463 (12)	0.9394 (2)	0.0838 (6)	
H9A	0.9256	0.2375	0.9852	0.101*	
C10	0.7497 (3)	0.17107 (10)	0.8523 (2)	0.0623 (5)	
C11	0.2638 (3)	0.09412 (11)	0.5796 (2)	0.0669 (6)	
C12	−0.2927 (3)	0.14545 (10)	0.4042 (2)	0.0772 (6)	
H12A	−0.2619	0.1895	0.3749	0.093*	
H12B	−0.2893	0.1495	0.5061	0.093*	
C13	−0.4652 (3)	0.12680 (12)	0.3005 (3)	0.0900 (7)	
H13A	−0.5412	0.1634	0.3004	0.108*	
H13B	−0.5001	0.0853	0.3366	0.108*	
C14	−0.4732 (3)	0.11527 (13)	0.1424 (3)	0.0995 (8)	
H14A	−0.5837	0.0996	0.0798	0.119*	
H14B	−0.4525	0.1583	0.1015	0.119*	
C15	−0.3471 (3)	0.06288 (13)	0.1397 (2)	0.0857 (7)	
H15A	−0.3759	0.0184	0.1690	0.103*	
H15B	−0.3490	0.0589	0.0383	0.103*	
C16	−0.1763 (3)	0.08263 (11)	0.2440 (3)	0.0852 (7)	
H16A	−0.0979	0.0471	0.2439	0.102*	
H16B	−0.1434	0.1251	0.2097	0.102*	

### Atomic displacement parameters (Å^2^)

**Table:**

	*U*^11^	*U*^22^	*U*^33^	*U*^12^	*U*^13^	*U*^23^
N1	0.0655 (12)	0.0642 (11)	0.0731 (11)	−0.0064 (8)	0.0116 (9)	0.0044 (8)
O1	0.0755 (11)	0.0761 (10)	0.1167 (13)	0.0101 (8)	0.0186 (9)	−0.0260 (8)
O2	0.0942 (12)	0.0604 (9)	0.0768 (10)	−0.0154 (7)	0.0110 (8)	−0.0074 (7)
O3	0.0677 (11)	0.0869 (11)	0.1111 (13)	−0.0032 (9)	0.0121 (9)	−0.0172 (9)
C1	0.0774 (16)	0.0539 (11)	0.0683 (13)	−0.0059 (11)	0.0182 (12)	−0.0120 (9)
C2	0.0689 (15)	0.0538 (12)	0.0641 (12)	0.0031 (11)	0.0203 (11)	−0.0032 (9)
C3	0.0682 (14)	0.0483 (10)	0.0481 (10)	−0.0035 (9)	0.0176 (9)	0.0014 (8)
C4	0.0753 (15)	0.0505 (11)	0.0529 (11)	−0.0009 (10)	0.0211 (10)	−0.0031 (8)
C5	0.0656 (14)	0.0660 (13)	0.0545 (11)	0.0015 (11)	0.0192 (10)	−0.0012 (9)
C6	0.0789 (19)	0.0897 (16)	0.0979 (17)	0.0125 (14)	0.0187 (14)	−0.0205 (13)
C7	0.071 (2)	0.130 (2)	0.131 (2)	0.0165 (16)	0.0138 (17)	−0.0327 (19)
C8	0.0669 (19)	0.126 (2)	0.119 (2)	−0.0029 (16)	0.0122 (15)	−0.0265 (18)
C9	0.0794 (17)	0.0836 (15)	0.0786 (15)	−0.0114 (13)	0.0181 (13)	−0.0138 (12)
C10	0.0655 (14)	0.0637 (13)	0.0536 (11)	−0.0056 (10)	0.0174 (10)	−0.0023 (9)
C11	0.0755 (16)	0.0566 (13)	0.0604 (12)	−0.0084 (12)	0.0160 (11)	0.0031 (10)
C12	0.1010 (19)	0.0594 (13)	0.0748 (14)	−0.0006 (12)	0.0371 (14)	0.0028 (10)
C13	0.0788 (18)	0.0879 (16)	0.1040 (19)	0.0125 (13)	0.0351 (15)	0.0085 (14)
C14	0.0805 (18)	0.116 (2)	0.0847 (17)	0.0087 (15)	0.0112 (13)	0.0173 (15)
C15	0.0853 (19)	0.1038 (18)	0.0645 (13)	−0.0124 (14)	0.0239 (13)	−0.0070 (12)
C16	0.0800 (18)	0.0910 (16)	0.0915 (16)	−0.0034 (13)	0.0401 (14)	−0.0088 (13)

### Geometric parameters (Å, °)

**Table:**

N1—C12	1.487 (2)	C7—C8	1.403 (3)
N1—C16	1.482 (3)	C7—H7A	0.9300
N1—H1C	0.9601	C8—C9	1.348 (3)
N1—H1D	0.9600	C8—H8A	0.9300
O1—C2	1.357 (2)	C9—C10	1.417 (3)
O1—H1B	0.8200	C9—H9A	0.9300
O2—C11	1.244 (2)	C12—C13	1.498 (3)
O3—C11	1.276 (2)	C12—H12A	0.9700
C1—C2	1.363 (3)	C12—H12B	0.9700
C1—C10	1.400 (3)	C13—C14	1.509 (3)
C1—H1A	0.9300	C13—H13A	0.9700
C2—C3	1.417 (2)	C13—H13B	0.9700
C3—C4	1.372 (3)	C14—C15	1.502 (3)
C3—C11	1.498 (3)	C14—H14A	0.9700
C4—C5	1.404 (3)	C14—H14B	0.9700
C4—H4A	0.9300	C15—C16	1.494 (3)
C5—C6	1.404 (3)	C15—H15A	0.9700
C5—C10	1.416 (2)	C15—H15B	0.9700
C6—C7	1.353 (3)	C16—H16A	0.9700
C6—H6A	0.9300	C16—H16B	0.9700
			
C12—N1—C16	111.63 (16)	C1—C10—C9	122.6 (2)
C12—N1—H1C	109.7	C5—C10—C9	118.3 (2)
C16—N1—H1C	109.4	O2—C11—O3	123.8 (2)
C12—N1—H1D	108.9	O2—C11—C3	120.0 (2)
C16—N1—H1D	109.2	O3—C11—C3	116.16 (19)
H1C—N1—H1D	108.0	N1—C12—C13	110.18 (17)
C2—O1—H1B	109.5	N1—C12—H12A	109.6
C2—C1—C10	121.22 (18)	C13—C12—H12A	109.6
C2—C1—H1A	119.4	N1—C12—H12B	109.6
C10—C1—H1A	119.4	C13—C12—H12B	109.6
O1—C2—C1	118.97 (18)	H12A—C12—H12B	108.1
O1—C2—C3	120.30 (19)	C12—C13—C14	111.27 (19)
C1—C2—C3	120.74 (19)	C12—C13—H13A	109.4
C4—C3—C2	118.14 (18)	C14—C13—H13A	109.4
C4—C3—C11	120.27 (18)	C12—C13—H13B	109.4
C2—C3—C11	121.58 (19)	C14—C13—H13B	109.4
C3—C4—C5	122.53 (18)	H13A—C13—H13B	108.0
C3—C4—H4A	118.7	C15—C14—C13	110.99 (19)
C5—C4—H4A	118.7	C15—C14—H14A	109.4
C6—C5—C4	122.70 (19)	C13—C14—H14A	109.4
C6—C5—C10	119.1 (2)	C15—C14—H14B	109.4
C4—C5—C10	118.21 (18)	C13—C14—H14B	109.4
C7—C6—C5	120.9 (2)	H14A—C14—H14B	108.0
C7—C6—H6A	119.6	C16—C15—C14	111.0 (2)
C5—C6—H6A	119.6	C16—C15—H15A	109.4
C6—C7—C8	120.4 (2)	C14—C15—H15A	109.4
C6—C7—H7A	119.8	C16—C15—H15B	109.4
C8—C7—H7A	119.8	C14—C15—H15B	109.4
C9—C8—C7	120.3 (2)	H15A—C15—H15B	108.0
C9—C8—H8A	119.8	N1—C16—C15	110.30 (17)
C7—C8—H8A	119.8	N1—C16—H16A	109.6
C8—C9—C10	121.0 (2)	C15—C16—H16A	109.6
C8—C9—H9A	119.5	N1—C16—H16B	109.6
C10—C9—H9A	119.5	C15—C16—H16B	109.6
C1—C10—C5	119.13 (19)	H16A—C16—H16B	108.1

### Hydrogen-bond geometry (Å, °)

**Table:**

*D*—H···*A*	*D*—H	H···*A*	*D*···*A*	*D*—H···*A*
O1—H1B···O3	0.82	1.77	2.504 (2)	149
N1—H1C···O2^i^	0.96	1.83	2.783 (2)	173
N1—H1D···O3	0.96	1.75	2.709 (2)	173
C12—H12A···O1^ii^	0.97	2.40	3.336 (3)	161

Symmetry codes: (i) −*x*, −*y*, −*z*+1; (ii) *x*−1/2, −*y*+1/2, *z*−1/2.
